# Neoadjuvant Therapy for Locally Advanced Esophageal Cancers

**DOI:** 10.3389/fonc.2022.734581

**Published:** 2022-04-07

**Authors:** Runkai Huang, Zhenbin Qiu, Chunwen Zheng, Ruijie Zeng, Wanxian Chen, Simeng Wang, Enmin Li, Yiwei Xu

**Affiliations:** ^1^ Department of Biochemistry and Molecular Biology, Shantou University Medical College, Shantou, China; ^2^ Department of Clinical Laboratory Medicine, the Cancer Hospital of Shantou University Medical College, Shantou, China; ^3^ Guangdong Esophageal Cancer Research Institute, Shantou University Medical College, Shantou, China

**Keywords:** locally advanced esophageal cancers, neoadjuvant, chemoradiotherapy, chemotherapy, immunotherapy

## Abstract

Esophageal carcinoma is one of the most aggressive malignant diseases. At present, neoadjuvant chemotherapy and neoadjuvant chemoradiotherapy are regarded as the standard modalities for the treatments of locally advanced esophageal cancers based on several landmark trials. However, the optimal regimen, radiation dose, and surgical intervals are uncertain and the rate of recurrence after neoadjuvant therapy is high. Patients receiving neoadjuvant therapy and reaching a pathological complete response have been reported to have a better survival benefit and a fewer recurrence risk than those non-pathological complete responses. Nevertheless, less than half of patients will reach a pathological complete response after neoadjuvant therapy, and the methods to evaluate the efficacy after neoadjuvant therapy accurately are limited. Immune checkpoint inhibitors have been recommended for the treatments of advanced esophageal cancers. Recently, research has been beginning to evaluate the safety and efficacy of immunotherapy combined with neoadjuvant therapy. Here, we will review and discuss the development of the neoadjuvant therapy of locally advanced esophageal cancers and unsolved clinical problems.

## Introduction

Esophageal carcinoma has been regarded as the seventh common cancer and the sixth leading cause of cancer death (1). Histologically, esophageal cancers include squamous cancer, which is common in Asian countries, and adenocarcinoma, which is common in western countries. To date, the treatment for esophageal cancers is still a tough clinical problem. For locally advanced esophageal cancers (LAECs), neoadjuvant chemotherapy (nCT) and neoadjuvant chemoradiotherapy (nCRT) have been recommended as standard treatments. Many studies have proved their anti-tumor efficacy, while some unsolved clinical problems also exist (2–4). Immune checkpoint inhibitors (ICIs) are being widely researched in various tumors. For esophageal cancers, ICIs have been recommended for the second/first-line treatment of advanced esophageal cancers. Recently, research regarding immunotherapy combined with conventionally neoadjuvant chemo(radio)therapy is ongoing. Hence, here we review and discuss the development of neoadjuvant therapy for LAECs and potential clinical problems.

## Neoadjuvant Chemotherapy

The research regarding the efficacy of nCT for LAECs has been conducted since the 1980s. As we summarized in **Table 1**, for adenocarcinoma, the utilization status of neoadjuvant treatments is owed to three randomized controlled trials. The UK Medical Research Council (OE02) trial was the first large-sized study to demonstrate a survival benefit of nCT for patients with esophageal cancer, in which patients were randomly assigned to receive preoperative chemotherapy or surgery alone, showing a 5% increase in 5-year survival for patients with adenocarcinoma (6, 7). Thus, nCT became a standard treatment for local esophageal adenocarcinoma (EAC). Besides, the Medical Research Council Adjuvant Gastric Infusional Chemotherapy (MAGIC) and Fédération Nationales des Centres de Lutte Contre le Cancer/Fédération Francophone de Cancérologie Digestive trials (FNCLCC-FFCD), which included 26% and 75% LAEC in the group receiving perioperative epirubicin, cisplatin, fluorouracil and cisplatin, fluorouracil respectively, showed that the perioperative chemotherapy group had a higher overall survival as shown by a hazard ratio (HR) reduction from 25% to 31% and a 5-year survival increase from 13% to 14% (8, 9). Since the benefit of survival, perioperative chemotherapy has become a standard treatment for locally gastroesophageal carcinoma. Recently, results from the trial 5-FU, Leucovorin, Oxaliplatin and Docetaxel (FLOT) Versus Epirubicin, Cisplatin and 5-FU (ECF) in Patients With Locally Advanced Resectable Gastric Cancer (FLOT4-AIO) revealed that the regimen with docetaxel, oxaliplatin, leucovorin, and fluorouracil showed better survival and disease control. Compared with perioperative epirubicin, cisplatin, and fluorouracil/capecitabine, the median overall survival (mOS) in patients administered with the FLOT regimen increased by 15 months (*P*=0.012) and the median disease-free survival (mDFS) increased by 12 months (*P*=0.0036) (10, 11). Thus, the FLOT has been one of the standard regimens for perioperative chemotherapy.

**Table 1:** Characteristics and main outcomes of clinical trials regarding neoadjuvant chemotherapy.

Trials	Histology	Interventions	Size	R0(%)	P value	pCR(%)	mOS (m)	P value	x-year OS (x,%)	P value
RTOG8911 (5) (1998)	ESCC, AC of esophgus, GEJ	Pre and Post: 3 and 2 cycles of cisplatin, 5-FU	213	62	NS	2.5	14.9	0.53	3, 23	NA
		Surgery	227	59			16.1		3, 26	
OE02 (6, 7) (2002)	ESCC, EAC	Pre: 2 cycles of cisplatin, 5-FU	390	60	<0.0001	4	16.8	0.004	5, 23	0.03
		Surgery	397	53			13.3		5, 17	
MAGIC (8) (2006)	AC of esophgus, GEJ, stomach	Pre and Post: 3 cycles of epirubicin, cisplatin, 5-FU	250	79.3	0.03	NA	NA	0.009	5, 36	NA
		Surgery	253	70.3			NA		5, 23	
FNCLCC-FFCD (9) (2011)	AC of esophgus, GEJ, stomach	Pre and Post: 2/3 and 3/4 cycles of cisplatin, 5-FU	113	87	0.04	NA	NA	NA	5, 38	0.02
		Surgery	111	74			NA		5, 24	
FLOT4-AIO (10, 11) (2016,2019)	AC of stomach or GEJ	Pre and Post: 4 cycles of docetaxel, oxaliplatin, leucovorin, and 5-FU	128	85	0.02	16	50	0.012	5, 45	NA
		Pre and Post: 3 cycles of epirubicin, cisplatin, 5-FU/capecitabine	137	74		6	35		5, 30	
JCOG9907 (2) (2015)	ESCC	Pre: 2 cycles of cisplatin, fluorouracil	164	96	0.04	5	NA	0.01	5, 55	0.04
		Post: 2 cycles of cisplatin, fluorouracil	166	91			NA		5, 43	

RTOG9811, Radiation Therapy Oncology Group trial 8911 trial; OE02, the UK Medical Research Council OE02 trial; MAGIC, the Medical Research Councial Adjuvant Gastric Infusional Chemotherapy trial; FNCLCC-FFCD, the Fédération Nationales des Centres de Lutte Contre le Cancer/Fédération Francophone de Cancérologie Digestive trial; JCOG9907, the Japan Clinical Oncology Group 9907 trial; FLOT4-AIO 5-FU, leucovorin, oxaliplatin, and docetaxel (FLOT) versus epirubicin, cisplatin, and 5-FU (ECF) in patients with locally advanced resectable gastric cancer; EAC, esophageal adenocarcinoma; ESCC, esophageal squamous carcinoma; AC, adenocarcinoma; GEJ, gastroesophageal junction; Pre, preoperative; Post, postoperative; 5-FU, 5-fluorouracil; pCR, pathological complete response; mOS, median overall survival; m, months; NA, not available.

For esophageal squamous carcinoma (ESCC), in the OE02 trial, 247 patients with ESCC were randomly assigned to receive nCT or surgery alone (6). Long-term results showed a benefit survival outcome (25.5% vs. 17.0% in 5-year OS) (7). The standard preoperative chemotherapy for locally advanced ESCC is cisplatin-fluorouracil based, which improved the R0 resection and overall survival (OS) in the Japan Clinical Oncology Group (JCOG) 9907 trial showing a 5-year OS of 55% in the nCT group while 43% in the postoperative treatment group (*P*=0.04). Based on this trial, the nCT based on cisplatin–fluorouracil (CF) has become the standard treatment for locally ESCC in Japan (2).

In summary, compared with surgery alone, adding chemotherapy before surgery has been proved to improve R0 resection rate and survival for patients with LAEC. For adenocarcinoma, the 5-year OS increased by 13%–15% by adding nCT. For ESCC, evidence from large-scale clinical trials is limited. The efficacy of nCT was confirmed in the JCOG9907 trial by comparing it with postoperative therapy (2). Nevertheless, the efficacy of nCT remains unsatisfactory as shown by a low pathological complete response (pCR) rate after nCT. For adenocarcinoma, thanks to the FLOT regimen, the pCR rate has been significantly improved, showing an increase of up to 16% (10). For ESCC, the pCR rate after a preoperative CF-based treatment was only 5%. Furthermore, although the JCOG9907 trial showed an OS benefit after nCT compared with postoperative therapy, there were no differences in progression-free survival (PFS; HR 0.84, 95%CI 0.63–1.11) indicating no improvement in the quality of life. Subgroup analysis reported that patients at stage III had less benefit than those at stage II. Thus, a more effective regimen is needed (2). Recent research has shown the promising efficacy of the regimen with docetaxel, cisplatin, and 5-FU (DCF). Results from a phase II trial reported a 90.5% completion rate and 17% pCR rate suggesting the feasibility of the DCF regimen (12). For disease control, the DCF regimen showed a higher 5-year PFS than that in the CF regimen (38.2% vs. 58.3%, *P*=0.006) (13). For security, the DCF regimen had no effects on surgical outcomes (14). However, the incidence of blood adverse events (AEs) was high (grade ≥3 neutropenia: 83%) (12). A propensity score-matched analysis reported that the DCF showed better efficacy for patients with locally advanced squamous carcinoma at stage III [objective response rate (ORR): 61.0% vs. 43.2%, *P*=0.021; HR for death 0.49, 95%CI 0.24–0.999, *P*=0.050] (15). Most recently, the JCOG1109 NExT trial (UMIN000009482), aiming to compare the efficacy between the CF regimen, the DCF regimen, and CF combined with radiotherapy in patients with locally advanced ESCC, reported its results in the 2022 American Society of Clinical Oncology Gastrointestinal Cancers Symposium (ASCO-GI) meeting, demonstrating that the DCF regimen was superior to the CF regimen in both OS and PFS with a high pCR rate of 19.8% and a manageable toxicity profile (mOS: 4.6 years versus not reach; median progression-free survival (mPFS): 2.7 years versus not reach). Based on this result, the DCF might be a new standard neoadjuvant treatment for locally advanced ESCC (16). In addition, there are other regimens for nCT (**Table 2**). However, it should be pointed out that there is a lack of large size, head-to-head clinical trials to compare the efficacy between nCT regimens. Besides exploring new regimens, the exploration of new modalities has also attracted much attention, like the combination of chemotherapy and immunotherapy, which will be shown below.

**Table 2:** Main outcomes of studies regarding different regimens used in neoadjuvant chemotherapy.

Regimen	Phase	Target	Interventions	Size	Rate of completion (%)	pCR (%)	Major adverse events (Grades 3–4)
CF (2)	III	ESCC	2 cycles cisplatin, 5-FU	164	85.4	5	Leukopenia 3% Thrombocytopenia 1% Diarrhea 1% Mucositis 3%
DCF (12)	II	ESCC	3 cycles of docetaxel, cisplatin,5-FU	42	90.5	17	Leukopenia 45.2% Neutropenia 83.3% Anorexia 7.1%
ACF (17)	II	ESCC	2 cycles of adriamycin, cisplatin, and 5-FU	81	88	0.00	Neutropenia 69% Leukopenia 58% Febrile neutropenia 17%
DOS (18)	I	Locally advanced AEG; advanced AEG, without distant metastasis	Level 1: 2 cycles of docetaxel, cisplatin,S-1 Level 2: 2 cycles of docetaxel, cisplatin, S-1	12	100	25	level 1: Neutropenia 50% Leukopenia 17% Febrile neutropenia 17% Level 2: Neutropenia 83% Leukopenia 50%
DCS (19)	II	ESCC of the thoracic esophagus	3 cycles of docetaxel, cisplatin, S-1	58	90	10	Leukopenia 50% Neutropenia: 68% Fbrile neutropenia: 18% hyponatremia 23%
DNF (20)	II	ESCC	3 cycles of docetaxel, nedaplatin, 5‐FU	28	89.30	32	Neutropenia 39.3% Leukocytopenia 32.1% Febrile neutropenia 10.7%
ECX (21)	III	EAC	4 cycles of epirubicin, cisplatin, capecitabine	446	81	Local pathology assessment: 11 Central pathology assessment: 7	Neutropenia 23% Diarrhea: 8% Nausea: 6% Vomiting: 6%
DNS (22)	II	ESCC	2 cycles of docetaxel, nedaplatin, S-1	32	96.90%	15.60%	Neutropenia 25% Leukopenia 18.8% Mucositis 15.6% Hyponatremia 15.6%

pCR, pathological complete response; ESCC, esophageal squamous carcinoma; AEG, adenocarcinoma of esophagogastric junction; EAC, esophageal adenocarcinoma.

## Neoadjuvant Chemoradiotherapy

Preoperative chemoradiotherapy plus surgery has been widely used for LAECs (**Table 3**). At the early stage, the results from clinical research were controversial since the heterogeneity between the studies included chemoradiotherapy regimens, surgical technique, tumor histology, sample size, and less advanced diagnostic methods. Until the ChemoRadiotherapy for Oesophageal cancer followed by Surgery Study (CROSS) trial and the Phase III Study of Neo-adjuvant Chemoradiotherapy Followed by Surgery for Squamous Cell Esophageal Cancer (NEOCRTEC5010) trial, the standard treatment status of nCRT for LAEC was established.

**Table 3:** Characteristics and main outcomes of clinical trials regarding neoadjuvant chemoradiotherapy versus surgery.

Trials	Histology	Regimen	Size	R0 (%)	P-value	pCR (%)	mOS (m)	P-value	x-year OS (x,%)	P-value
Walsh (23) (1996)	EAC	2 cycles of cisplatin, 5-FU+40 Gy	58	NA	NA	25	16	0.01	3, 32	0.01
		Surgery	55	NA			11		3, 6	
Bosset (24) (1997)	ESCC	2 cycles of cisplatin+37 Gy	143	81.2	0.017	26	18.6	0.78	NA	NA
		Surgery	139	68.6			18.6		NA	
Burmeister (25) (2005)	ESCC, EAC	Cisplatin, 5-FU+35 Gy	128	80	0.0002	16	22.2	0.44	NA	NA
		Surgery	128	59			19.3		NA	
GALGB9781 (26) (2008)	ESCC, EAC, AC of GEJ	Cisplatin+5-FU+50.4 Gy	30	NA	NA	40	4.48 y	0.002	5, 39	NA
		Surgery	26	NA			1.79 y		5, 16	
CROSS (3) (2012)	ESCC, EAC, AC of GEJ	5 cycles of carboplatin, paclitaxel+41.4 Gy	188	92	<0.001	29	49.4	0.003	5, 47	NA
		Surgery	180	69			24		5, 34	
NEOCRTEC 5010 (4) (2018)	ESCC	2 cycles of vinorelbine, cisplatin +40.0 Gy	224	98.4	0.002	43.2	100.1	0.025	3, 69.1	NA
		Surgery	227	91.2			66.5		3, 58.9	

GALGB9781, Cancer and Leukemia Group B 9781; CROSS, the ChemoRadiotherapy for Oesophageal cancer followed by Surgery Study trial; NEOCRTEC5010 Phase III Study of Neo-adjuvant Chemoradiotherapy Followed by Surgery for Squamous Cell Esophageal Cancer; EAC, esophageal adenocarcinoma; ESCC, esophageal squamous carcinoma; AC, adenocarcinoma; GEJ, gastroesophageal junction; pCR, pathologic complete response; mOS, median overall survival; m, months; y, years; NA, not available.

For adenocarcinoma, in the CROSS trial, 336 patients, in which EAC accounted for 75%, were enrolled to receive nCRT or surgery alone. Initial results showed that after nCRT, the resection specimen demonstrated that a pCR was 23% and there were no differences in postoperative complications and in-hospital mortality between the nCRT group and the surgery group, which meant the feasibility and acceptable toxicity of the CROSS regimen (3). Long-term results revealed a benefit of OS [mOS: 43.2 vs. 21.7 months (m)] and PFS (mPFS: 29.9 vs. 17.7 m), and the effect of OS was up to 10 years of follow-up, indicating that compared with surgery, the nCRT based on the CROSS regimen significantly prolonged lifespans and improved disease control (27, 28). Thus, nCRT was regarded as the standard treatment for locally EAC and the regimen; 5 cycles of paclitaxel (50 mg/m^2^) and 3 cycles of carboplatin (2 mg/AUC) concurrent with 41.4Gy became the standard regimen for nCRT.

For ESCC, however, although the CROSS trial reported a much better efficacy of nCRT (pCR rate: 49%; mOS: 81.6 m:21.1 m; mPFS: 74.7 m: 11.6 m; 10-year OS rate: 46% vs. 23%), it should be noted that only 23% tumor types were ESCC, which might make it hard to convince the benefit of ESCC (3, 27, 28). Most recently, the results from the NEOCRTEC5010 trial demonstrated the better efficacy of nCRT (vinorelbine, cisplatin, 40.0 Gy) versus surgery alone. The R0 rate was significantly higher in the nCRT group than that in the surgery group. Resection specimens showed that the pCR rate was 43.2%. With a median follow-up of 41.0 months, significant differences in mOS and 3-year OS were found in favor of nCRT (mOS: 100.1 vs. 66.5 m, *P*=0.025; 3-year OS: 69.1% vs. 58.9%). Furthermore, disease-free survival (DFS) was also significantly improved in the nCRT group compared with the surgery group (100.1 vs. 41.7 m, P<0.001) (4). Based on these two trials, nCRT also became the standard treatment modality for locally ESC and the regimen, 2 cycles of vinorelbine (25 mg/m^2^) and cisplatin (75 mg/m^2^) concurrent with 40.0 Gy became the standard regimen for nCRT in China.

In summary, most of the studies comparing the efficacy of nCRT to surgery alone obtained negative results before the 2000s. After the 2000s, many studies showed that nCRT was better than surgery alone. Based on the results from the landmark trials, CROSS and NEOCRTEC5010, nCRT has become the standard treatment for LAECs. The CROSS regimen has been widely used around the world. Current evidence shows that compared with surgery alone treatment, nCRT can ameliorate R0, pCR, OS, and recurrence. After nCRT, R0, pCR, and OS range from 81% to 98%, 25% to 43%, and 16 to 100.1 months, respectively.

## Neoadjuvant Chemoradiotherapy vs. Neoadjuvant Chemotherapy

As mentioned above, nCT and nCRT are two main modalities for the treatment of LAECs. In Japan, based on its own research, nCT was the standard modality for LAECs. In some western countries, nCRT was the preferred treatment modality based on the results of the CROSS trial. Based on the NEOCRTEC5010, nCRT also has become the standard treatment for locally ESCC in China. Whether nCRT or nCT brings better efficacy for patients with LAECs is still uncertain so far. Research regarding the comparison between nCT versus nCRT directly is limited and the current evidence is inconclusive. Current research demonstrated that whether in ESCC or EAC, patients receiving nCRT are more likely to reach pCR. The pCR rate in nCRT is higher than that in nCT. However, there are no differences between these two modalities in survival outcomes.

For EAC, only three prospective studies directly compared the advantages and disadvantages of the two modalities. In the PreOperative therapy in Esophagogastric Adenocarcinoma (POET) trial, patients after nCRT showed a significantly higher pCR rate of 14.3% compared with 1.9% after nCT. For OS, there was a trend in favor of nCRT as shown by a longer mOS and higher rate of long-term survival (29, 30). In the Neoadjuvant Chemotherapy Versus Radiochemotherapy for Cancer of the Esophagus or Cardia (NeoRes) trial, which enrolled over 70% of patients with EAC, the authors demonstrated a significant increase of 19% of pCR in the nCRT setting but no differences for OS compared with nCT (31, 32). Also, Burmeister et al. reported similar results that the pCR rate in nCRT significantly increased, while mOS and the long-term survival rate were only higher numerically (33).

The research about the efficacy of nCRT versus nCT in ESCC is limited (**Table 4**). The results from the NeoRes trial containing less than 30% of patients with ESSC could not find benefit in long-term survival although the pCR rate after nCRT was significantly higher than after nCT. The trial, Comparison Between NCRT and NCT Followed by MIE for Treatment of Locally Advanced Resectable ESCC (CMISG1701), reported its initial results, which were to compare the efficacy of four cycles of paclitaxel (50 mg/m^2^)/cisplatin(25 mg/m^2^) as nCRT regimen, followed by minimally invasive esophagectomy (MIE) versus two cycles of paclitaxel (135 mg/m^2^)/cisplatin(75 mg/m^2^) as nCT regimen followed MIE in ESCC. The study showed that patients undergoing nCRT had better pathologic outcomes including higher pCR (35.7% vs. 3.8% P<0.01) and less lymph nodes involved (66.1% vs. 46.2%, P = 0.03). However, 1-year OS was not different between the two groups (34).

**Table 4:** Characteristics and outcomes of studies regarding neoadjuvant chemotherapy vs. neoadjuvant chemoradiotherapy.

Studies	Target	Regimen	Size	R0 (%)	pCR (%)	OS (m)	3-year OS (%)	5-year OS (%)
				Neoadjuvant chemotherapy vs. neoadjuvant chemoradiotherapy				
Michael Stahl (29, 30) (2009, 2017)	AC of GEJ	**nCT:** 2.5 cycles of 5-FU,leucovorin,cisplatin **nCRT:** 2 cycles of 5-FU, leucovorin, cisplatin followed by cisplatin, etoposide+30 Gy	119	69.5 vs. 72 NS	1.9 vs. 14.3 P=0.03	21.1 vs. 30.8 m P=0.055	26.1 vs. 46.7	24.4 vs. 39.5
NeoRes (31, 32) (2016,2018)	ESCC, EAC, AC of GEJ	**nCT:** 3 cycles of cisplatin, 5-FU **nCRT:** 3 cycles of cisplatin, 5-FU+40 Gy	181	74 vs. 87 P=0.04	9.0 vs. 28.0 P=0.002	31.4 vs. 36.0 m	49 vs. 47 P=0.77	39.6% vs. 42.2 P=0.60
Bryan H. Burmeister (33) 2011	EAC, AC of GEJ	**nCT:** 2 cycles of cisplatin, 5-FU **nCRT:** 2 cycles of cisplatin, 5-FU+35 Gy	75	80.5 vs. 84.6 P=0.61	0 vs. 13 P=0.02	29 vs. 32 m P=0.83	49 vs. 52 P=0.97	36 vs. 45 P=0.60
Hao Wang (34) 2021	ESCC	**nCT:** 2 cycles of paclitaxel, cisplatin **nCRT:** 4 cycles of paclitaxel, cisplatin+40 Gy	264	96.2 vs. 97.3 P=0.92	3.8 vs. 35.7 P<0.01	NA	1-year OS 82.6 vs. 87.1 P = .30	NA

NeoRes, the Neoadjuvant Chemotherapy Versus Radiochemotherapy for Cancer of the Esophagus or Cardia trial; AC, adenocarcinoma; GEJ, gastroesophageal junction; EAC, esophageal adenocarcinoma; NA, not available; nCT, neoadjuvant chemotherapy; nCRT, neoadjuvant chemoradiotherapy..

As the lack of evidence of direct comparison, meta-analyses were conducted. A network meta-analysis including 26 studies compared the efficacy of surgery alone, nCT, neoadjuvant radiotherapy, nCRT, surgery followed by adjuvant chemotherapy, adjuvant radiotherapy, or adjuvant chemoradiotherapy. A ranking analysis reported that nCRT might be the best option for patients diagnosed with LAECs. When compared to surgery alone, in all the treatments, nCRT yielded the best benefit in terms of OS and PFS/DFS (HR = 0.76, 95%CI 0.67–0.85; HR = 0.8, 95%CI 0.68–0.94, respectively). Also, only nCRT associated with a statistically confident decrease in locoregional recurrence or distant metastasis [odd ratios (OR)=0.48, 95%CI 0.30–0.77; OR = 0.67, 95%CI 0.49–0.93, respectively] (35).

To sum up, currently, it is still unable to define which modality is better for LAECs. Current evidence suggests that despite the histological type, patients with LAECs are more likely to develop pCR after nCRT, whereas in the OS of nCRT, there was no statistical improvement compared to that of nCT. Reasons why the higher rate of pCR after nCRT fails to translate into the benefit of survival, are still a primary concern. The toxicities of treatments and perioperative complications may contribute to the problem. The POET trial showed that the grade 3/4 toxicities were 5% in the nCT group, while grade 3/4 leukocytopenia and thrombocytopenia were 12% and 5%, respectively, in the nCRT group (29). The NeoRes trial demonstrated that the severity of postoperative complications in the nCRT group was significantly higher than that in the nCT group (*P*=0.001) (31). Long-term results showed that the patients after nCRT had higher risks of postoperative complications (9% vs. 1% *P*=0.02) (32). Meta-analyses also reported a significantly higher postoperative mortality in nCRT (RR 1.58, 95% CR 1.00– 2.49) (36). We hypothesize that in looking for new drugs or treatment modalities that can lead to fewer toxicities, and the improvement of surgery technology, the introduction of early interdisciplinary supportive care (ESC) may help solve the problem. Most recently, a phase III clinical trial explored whether ESC combined with the standard first-line treatment for patients with metastatic esophageal cancers could improve the prognosis. Results showed the mOS in the ESC group was significantly higher than those in the standard care group (14.8 vs. 11.9 m, HR 0.68, 95%CI 0.51–0.9, *P*=0.021) (37). Another study demonstrated that a multidisciplinary team approach started before neoadjuvant therapy would decrease the risk of AE rate during chemotherapy (*P*=0.007) and provide safe perioperative conditions(*P*=0.003) (38). MIE is becoming more and more common in surgical treatments. A new meta-analysis has reported that MIE decreased 18% risk of all-cause 5-year mortality for patients with esophageal cancers compared with open surgery (39). Nowadays, the regimens of neoadjuvant therapy are various. CROSS, MAGIC, and FLOT regimens have been widely used. Here, we summarize the ongoing clinical trials comparing the efficacy of nCRT versus nCT based on these regimens, expecting that more valid and powerful evidence can be provided by these trials (**Table 5**).

**Table 5:** Characteristics clinical trials regarding neoadjuvant chemotherapy vs. neoadjuvant chemoradiotherapy.

Clinical trials	Phase	Size	Conditions	Interventions	Endpoints		Status
					Primary	Secondary	
NCT01404156	II/III	60	EAC, AEG	**nCT:** FLOT/ECF/ECX regimen + surgery **nCRT:** CROSS regimen concurrently with 45 Gy + surgery	Compliance to treatment, Treatment response	3-year OS, DFS QoL	Recruiting
NCT02509286	III	438	EAC, AEG	**nCT:** FLOT **nCRT:** CROSS regimen concurrently with 41.4 Gy + surgery	OS	PFS, site of failure, RFS, QoL, complications	Not recruiting
NCT01726452	III	377	EAC, AEG	**nCT:** modified MAGIC/FLOT + surgery **nCRT:** CROSS regimen concurrently with 41.4 Gy + surgery	OS	None	Not recruiting
UMIN000009482	III	600	ESCC	**nCT:** 2 cycles of cisplatin,5-FU or docetaxel, cisplatin, 5-FU +surgery **nCRT:** 2 cycles of cisplatin, 5-FU concurrently with 41.4 Gy + surgery	OS	PFS, R0, pCR, AE, morbidity, toxicity, response rate	No longer recruiting
NCT03001596	NA	264	ESCC	**nCT:** 2 cycles of paclitaxel, cisplatin+MIE **nCRT:** 4 cycles of paclitaxel, cisplatin concurrently with 40 Gy+MIE	OS	PFS, R0, RFS, QoL, pathological response rate, treatment-related complication, positive lymph nodes’ number	Completed
NCT04138212	III	456	ESCC	**nCT:** 2 cycles of paclitaxel, cisplatin + surgery **nCRT:** CROSS regimen concurrently with 41.4 Gy + surgery	OS	DFS, pCR, AE, complications	Recruiting
NCT03579004	II	48	ESCC	**nCRT:** 2 cycles of paclitaxel, cisplatin+ paclitaxel, cisplatin concurrently with 44 Gy+surgery	DFS	OS, ORR, pCR, number of treatment-related AEs	Unknown
NCT03013010	III	682	AC of stomach, GEJ	**nCT:** 3 cycles of S-1, oxaliplatin+ surgery **nCRT:** 1 cycle of S-1, oxaliplatin +5 weeks of S-1and oxaliplatin concurrently with 45 Gy+surgery+3 cycles of S-1 and oxaliplatin	DFS	OS, R0, toxicity, complications, pathological response rate	Recruiting

EAC, esophageal adenocarcinoma; AEG, adenocarcinoma of esophagogastric-junction; nCT, neoadjuvant chemotherapy; nCRT, neoadjuvant chemoradiotherapy; AC, adenocarcinoma; GEJ, gastroesophageal junction; ESCC, esophageal squamous carcinoma; MIE, minimally invasive esophagectomy; OS, overall survival; DFS, disease-free survival; PFS, progression-free survival; RFS, recurrence-free survival; pCR, pathological response rate; QoL, quality of life; AE, adverse events; RFS, recurrence-free survival time; ORR, objective response rate; NA, not applicable. FLOT 4 cycles of 5FU (2,600 mg/m^2^), leucovorin (200 mg/m^2^), oxaliplatin (85 mg/m^2^), and docetaxel (50 mg/m^2^) on day 1, q2w perioperatively, ECF/ECX 3 cycles of epirubicin (50 mg/m^2^) on day 1 cisplatin (60 mg/m^2^),5-FU (200 mg/m^2^)/capecitabine (625 mg/m^2^) for 21 days perioperatively, CROSS 5 cycles of paclitaxel (50 mg/m^2^) and carboplatin (2 mg/AUC) on days 1, 8, 15, 22, and 29, modified MAGIC 3 cycles of epirubicin (50 mg/m^2^) on day 1, cisplatin (60 mg/m^2^) on day1/oxaliplatin (139 mg/m^2^) on day 1, 5-FU (200 mg/m^2^) for 21 days/capecitabine (625 mg/m^2^) perioperatively.

## Problems in Neoadjuvant Chemoradiotherapy

For nCRT, however, there are some problems to be solved, which are shown as follows.

Nowadays, the optimal regimen for nCRT is still inconclusive. The excellent efficacy of nCRT showed in clinical trials cannot be reproduced completely in a real-world scenario (**Table 6**). Four studies that evaluated the efficacy of the CROSS regimen in the real-world scenario demonstrated that patients who did not fully meet the CROSS criteria had a lower efficacy than those who fully met them, showing a lower pCR and mOS and possibly higher postoperative morbidity and mortality (40–43). Moreover, even patients eligible for the criteria could not obtain the efficacy as well as that in the CROSS trial. Based on the results of the CROSS trial, the CROSS regimen has replaced the CF-based regimen that was used widely before. However, no prospective head-to-head comparative study was conducted. A propensity score-matched study, comparing the efficacy of the cisplatin/fluorouracil regimen versus the CROSS regimen in patients with locally advanced ESCC, recently reported that there were no differences in the pathological or survival outcome between the two regimens but the study showed a trend in favor of the cisplatin/fluorouracil regimen (44). For adenocarcinoma, one retrospective study (adenocarcinoma: 86%) showed that the CF regimen could increase the pCR rate (*P*=0.032), improve the recurrence-free survival (HR 0.39, 95%CI 0.21–0.73, *P*=0.003) and OS (HR 0.46, 95%CI 0.24–0.87, *P*=0.016) (45). In addition, retrospective research, comparing the NEOCRTECT5010 regimen to the CF regimen, demonstrated the former increased pCR rate (47.4% vs. 28.1%, *P*=0.034) and contributed to better OS (52.8 vs. 25.2 m, *P*=0.001) while leading to increasing hematologic toxicities (*P*=0.03) (46).

**Table 6:** Comparison of efficacy of CROSS regimen in real-world scenario versus clinical trial.

Indexes	CROSS-eligible	Extended-CROSS	P-value	CROSS trial`
R0	95.8%^1^ 83.3% for ESCC^2^	95.2%^1^ 84% for ESCC^2^	0.406^1^ 0.959^2^	92%
pCR	16.8% for EAC^1^ 48.2% for ESCC^1^ 33.3% for ESCC^2^ 27% for overall^3^	16.9 for EAC^1^ 33.3 for ESCC^1^ 20% for ESCC^2^ 28% for overall^3^	0.908 for EAC^1^ 0.000 for ESCC^1^ 0.253^2^ 0.76^3^	29% for overall 23% for EAC 49% for ESCC
mOS	24.2 m for ESCC^2^ 58.5 m for overall^3^ 37.3 m for overall^4^	12.7 m for ESCC^2^ 35.0 m for overall^3^ 17.2 m for overall^4^	0.047^2^ 0.90^3^ 0.004^4^	48.6 m for overall 43.2 m for EAC 81.6 m for ESCC
Postoperative mortality (<30 d)	3.2%^1^ 0^2^ 3%^3^ 2.2%^4^	4.6%^1^ 0^2^ 3%^3^ 4.2%^4^	0.037^1^ 1.00^3^ 0.486^4^	2%
Postoperative morbidity	58.3%^1^ 16.7% for anastomotic leakage^2^ 2.8% for chylothorax^2^ 5.6% for fistula^2^ 2.8 for ischemic conduit^2^ 22.2 for cardiac complications^2^ 19.4% for pulmonary complications^2^ 2.8% for urinary complications^2^ 19.4% for vocal cord palsy^2^ 64%^3^	61.8%^1^ 12% for anastomotic leakage^2^ 4% for ischemic conduit^2^ 4% for mediastinitis^2^ 24% for cardiac complications^2^ 16% for pulmonary complications^2^ 8% for urinary complications^2^ 12% for vocal cord palsy^2^ 63%^3^	0.048^1^ NS^2^ 0.83^3^ NS^4^	46% for pulmonary complications 21% for cardiac complications 10% for chylothorax 3% for mediastinitis 22% for anastomotic leakage

pCR, pathological complete response; mOS, median overall survival; ESCC, esophageal squamous carcinoma; EAC, esophageal adenocarcinoma, ^1^ (40), ^2^ (41), ^3^ (42), ^4^ (43).

The radiation dose is various in nCRT ranging from 37 to 50.4 Gy. A retrospective study evaluated the efficacy of high dose (>45Gy) versus low dose (≤45 Gy) in ESCC, showing no differences in pCR rate and survival (47). Recently, a systematic review incorporated 110 studies, involving ESCC/EAC, where patients receiving nCRT up to a dose of 50.4 Gy, to evaluate the efficacy and safety of different radiation doses and try to find an optimal dose. Results demonstrated that 40.0–41.4 Gy might be a rational dose for nCRT (48). Prospective controlled studies are needed to confirm the optimal dose.

So far, the surgical interval for nCRT is 4–6 weeks. The optimal surgical interval for nCRT is still inconclusive. The proper extension of the surgical interval may increase the pCR rate because of the shrinkage of tumors under the effects of nCRT. Also, it gives patients more time to recover from the preoperative treatments, which may reduce the risk of surgical-related AEs. All these may bring a benefit of survival. However, some research reported the increase in pCR rate profiting from the extending surgical interval failed to translate into a benefit of survival (49, 50). There may be several reasons. First, these studies are retrospective studies where patients delayed surgeries because of their poor body conditions or the AEs from nCRT rather than their preferences. Second, although extending surgical interval increases the pCR rate, its contribution is not enough to reflect on a statistically significant benefit in survival. Subgroup analysis, comparing the efficacy of extension of surgical interval between patients reaching pCR versus non-pCR, showing a significantly better survival benefit supported this point (8.7 years for patients with pCR vs. 2.0 years) (49). Recently, one prospective research, avoiding the gap of the first reason, reported that the extension of the surgical interval had no effects on short-term operative outcomes (overall postoperative complication: 63.2% vs. 72.6%, *P*=0,134; severe postoperative complications: 31.6% vs. 34.9%; median length of hospital stays: 15 vs. 17 d, *P*=0.234) (51). More prospective studies are needed to focus on the effect of surgical interval on survival.

Although nCRT improves the recurrences of patients compared with surgery, the recurrent rate after nCRT is still high. The ten-year outcome of the CROSS trial revealed that recurrences occurred in 48% of patients, and 33.7% of patients reported in the NEOCRTEC5010 trial (4, 28). Most recurrences occurred in the first 3 years after surgery. Compared with distant metastases, nCRT mainly improved local or regional recurrences. Data from the ten-year outcome of the CROSS trial demonstrated that the overall local-regional recurrence rate after nCRT reduced significantly from 40% to 21% compared with surgery alone (28). Similarly, in the NEOCRTEC5010 trial, nCRT significantly improved the overall local–regional recurrence rate (14.1% vs. 22.5%, *P*=0.031) while the overall distant metastasis rate had no statistical differences (23.9% vs. 31.7%, *P*=0.08) *(* 52). Thus, distant metastasis is the main failed mode after nCRT. Subgroup analysis showed that patients who reached pCR had a lower recurrence rate than those with non-pCR. Histologically, the patients with ESCC or EAC who did not reach pCR after nCRT showed a different recurrent pattern. The patients with EAC showed a likelihood of recurrence compared with ESCC (43.2% vs. 34.3%, *P*=0.023). The patients with ESCC had a higher risk of regional and supraclavicular recurrences while a lower hematogenous metastasis compared with EAC. In addition, it was reported compared with EAC, patients with ESCC had a significantly higher rate of failure to receive salvage treatments (*P*=0.005) mainly because of poor performance status (53).

The high recurrence rate after nCRT demands a close monitor. Besides, salvage measures are essential when recurrence occurs. The differences in recurrence patterns between ESCC and EAC require different strategies based on histological tumor types. For EAC, distant metastasis was the main recurrence pattern, which means the necessity of the systematic treatment after nCRT. The Checkmate577 trial had reported that the addition of nivolumab as an adjuvant therapy could significantly reduce the risk of recurrences for patients with residual disease after nCRT (HR 0.74, 95%CI 0.60–0.92). The DFS in the nivolumab group was twice as long as that in the nCRT alone group (22.4 vs. 11.0 m, P<0.001) (54). For ESCC, local–regional recurrence was the main failed mode, indicating the need for the enhancement of local treatment. Systematic lymph node dissection has been recommended in the surgery alone for patients with esophageal cancers. However, whether patients with nCRT followed by systematic lymph node dissection can obtain a survival benefit is debatable. Most recently, a second analysis from the result of the NEOCRTEC5010 trial revealed that systematic lymph node dissection did not increase the surgical risk and could improve the survival and control of disease for patients with nCRT (mOS: 100.0 vs. 85.5 m, *P*=0.01; 3-year OS: 75.2% vs. 61.5%; 3-year DFS: 70.2% vs. 55.5%, *P*<0.001). Compared with the dissection of lymph node <20, the dissection of lymph node ≥20 brought a lower recurrence rate (25.8% vs. 41.2%, *P*=0.027) and better control of disease (5.2% vs. 18.8%, *P*=0.004) (55). Thus, systematic lymph node dissection should be recommended in nCRT for ESCC.

## Evaluation for the Efficacy of Neoadjuvant Setting

pCR is a strong predictor of a good prognosis after nCRT. Many studies have shown that patients reaching pCR after nCRT would obtain a longer survival and a lower risk of recurrences compared with those with non-pCR. Current evidence shows that after nCRT, the rate of pCR ranges from 20% to 43%, which means that a lot of patients still cannot benefit from nCRT. Furthermore, treatment-related toxicities and the extension of operation may lead to a poorer physical condition and tumor progression. Thus, the development of methods to evaluate a pathological response after nCRT is essential to improve the efficacy of nCRT and avoid unnecessary treatments.

The accuracy of using a single imaging method to find residual disease after nCRT is limited. Recently, a meta-analysis reported the limited accuracy of endoscopic biopsies, endoscopic ultrasound, and positron emission tomography with 2-deoxy-2-[fluorine-18]fluoro-D-glucose integrated (with computed tomography) (18F-FDG PET(-CT)) as single modalities to detect residual disease after nCRT for patients with LAECs (56).

Magnetic resonance imaging (MRI) has been reported to have a promising accuracy in the evaluation of efficacy after nCRT. A prospective study showed the relative increase of the parameter of diffusion-weighted magnetic resonance imaging (DW-MRI), ΔADC_during-pre_(median apparent diffusion coefficient; during 2–3 weeks during nCRT) was positively correlatively with pCR. A cut-off value of 29% yielded a sensitivity of 100%, specificity of 75%, accuracy of 95%, positive predictive value of 94%, and negative predictive value of 100% (57). The parameters of diffusion contrast-enhanced magnetic resonance imaging (DCE-MRI), ΔAUC (area under the concentration–time curve), were reported to predict pCR. At a cut-off of 24.6%, ΔAUC_post-pre_ mostly predicted a pCR, yielding a sensitivity of 83%, specificity of 88%, positive predictive value of 71%, and negative predictive value of 93% (58). Based on these two studies, another study evaluated the accuracy of the combination of DW-MRI and DCE-MRI and reported their complementary value (59). The addition of MRI into gastroscopy with biopsies and endosonographic ultrasound with fine-needle aspiration has been reported to improve the detection of residual tumor after nCRT, as shown by an increased sensitivity from 47% to 89% (60). A prospective study evaluated the combined value of DW-MRI and 18F-FDG PET/CT to predict pCR in patients after nCRT. Results showed that early changes of the parameter on DW-MRI during nCRT and changes on 18F-FDG PET/CT after nCRT might yield a complementary value in the assessment of pCR (61). The Surgery AS Needed for Oesophageal Cancer (preSANO) trial evaluated the efficiency of the combination of different methods to detect residual disease after nCRT and tried to propose an optimal modality. Results showed that endoscopic ultrasonography, bite-on-bite biopsies, and a fine-needle aspiration of suspicious lymph nodes to detect locoregional residual disease combined with PET-CT for the detection of interval metastases were an effective modality for clinical evaluation (62). Now, a phase III trial (NTR6803) has been conducted to compare the outcome of active surveillance with standard resection in patients who reached pCR by using this strategy. Based on the preSANO trial, Chinese scholars are evaluating this strategy in patients with locally advanced ESCC(NCT03937362). Another ongoing trial is evaluating the combined value of DW-MRI, DCE-MRI, 18F-FDG PET/CT, and circulating tumor DNA (ctDNA) to predict the pathological response (NCT03474341).

Compared with multi-imaging methods, a single-imaging method provides limited information. Moreover, usually, imaging methods like CT and endoscopic ultrasound mainly provide anatomical information. However, there are lots of biological parameters like tumor metabolism, structure, and function of blood vessels in tumor tissues, which are sensitive and change early when the tumor tissues react to clinical interventions. This may explain that the combination of multiple imaging methods and addition of MRI can improve the evaluation of pathological response after nCRT. In the future, more attention should be paid to the application of radiomics in the efficacy evaluation after nCRT.

Biomarkers, related to tumor growth, DNA repair, cell cycle, etc., have been investigated to see the predictive value in histology response after nCRT in LAECs. ERCC1 and p53 were probably studied widely. As results regarding the predictive value of p53 to pCR were debatable, Zhang et al. assembled 28 studies in their meta-analysis and reported that the wild-type form of p53 status was probably a predictive biomarker for pCR after nCRT (63). Other molecular markers like cyclinD, p53R2, COX-2, Gli-1, and miRNA also have been explored. Recently, one meta-analysis analyzed that 56 biomarkersm except for p53 from 46 articles, demonstrated that the low expression of COX2, miR-200c, ERCC1, and TS, or a higher expression of CDC258 and p16, were associated with the prediction of response for patients receiving neoadjuvant chemo(radio)therapy (64). However, there are still no effective biomarkers to predict whether someone will respond to chemoradiotherapy or not. The joining of imaging techniques and specimen detection (tissue and/or liquid) has rarely been reported yet, which may have synergistic effects on the evaluation or prediction of response to the neoadjuvant setting.

## Immunotherapy in Locally Advanced Esophageal Cancer

With the insight of the molecular mechanisms of tumors and highlight of individualized treatment, targeted therapy has emerged as a hot direction. The overexpression of Epidermal Growth Factor Receptor (EGFR), Erb-B2 Receptor Tyrosine Kinase 2 (HER2), and Vascular Endothelial Growth Factor Receptor (VEGFR) has been reported in esophageal cancer, which enhances tumor occurrence, progression, and drug resistance. Adding drugs targeting these molecules to the current treatment may bring a synergistic effect. However, so far, most studies failed to show a satisfactory efficacy (65, 66). In recent years, ICIs have been a new modality used in tumor treatments like none small cell lung cancer and melanoma because of their promising efficacy. For esophageal cancer, some research has reported the anti-tumor activity of ICIs. Three landmark clinical trials, Study of Pembrolizumab (MK-3475) Versus Investigator’s Choice Standard Therapy for Participants With Advanced Esophageal/Esophagogastric Junction Carcinoma That Progressed After First-Line Therapy (KEYNOTE181), Study of Nivolumab in Unresectable Advanced or Recurrent Esophageal Cancer (ATTRACTION-3), and Study of SHR-1210 Versus Investigator’s Choice Standard Therapy for Participants With Advanced Esophageal Cancer (ESCORT) have confirmed the anti-tumor activity of pembrolizumab, nivolumab, and camrelizumab in second-line treatment for advanced/metastatic esophageal cancer by demonstrating that these drugs improved survival, the ORR, and duration of response (DoR) compared with conventional chemotherapy (67–69). With the success in second-line treatments, the usage as first-line treatment in advanced/metastatic esophageal cancer is already being developed. A KEYNOTE-590 trial reported that pembrolizumab combined with a platinum-based regimen (cisplatin/5-FU) as the first-line treatment for advanced/metastatic esophageal cancers showed better survival outcomes compared with a platinum-based regimen alone. For safety, the two regimens were similar (70). A Checkmate-649 trial firstly compared the efficacy of nivolumab combined with capecitabine/oxaliplatin or leucovorin/fluorouracil/oxaliplatin to chemotherapy alone. Data showed that the ORR in patients receiving the new regimen was more than 50%. Compared with chemotherapy alone, the new regimen prolonged the DoR by 2.5 months, reduced the risk of death by 23%, increased the median OS by 2.2 months (71). The Study of SHR-1210 in Combination With Chemotherapy in Advanced Esophageal Cancer (ESCORT-1st) trial evaluated the efficacy of camrelizumab combined with paclitaxel/cisplatin as the first-line regimen for advanced/metastatic ESCC. Data demonstrated that compared with chemotherapy alone, camrelizumab combined with chemotherapy reduced the risk of death by 30% and 44% in the risk of progression. The median OS and PFS were prolonged by 2.1 and 1.3 months, respectively. The ORR rate was 72.1% in the new regimen group, and the DoR increased by 2.4 months. Moreover, the addition of camrelizumab did not increase the rate of AEs (72).

All these results revealed that immunotherapy combined with chemotherapy could improve survival with no unacceptable AEs for advanced/metastatic esophageal cancers. Notably, the higher ORR after immunotherapy combined with chemotherapy means more patients can reduce their tumor volume by 30% after the treatment. Tumor shrinking may lead to downstage, which may lay a foundation for the addition of ICIs into neoadjuvant therapy for LAECs (**Supplementary Table 1**).

For EAC, one trial (NCT03044613) evaluated the safety and efficacy of the induction therapy of nivolumab, followed by mivolumab concurrently with nCRT. Data suggested acceptable toxicities without the delay of surgery and a high pCR of 40% (73).

For ESCC, one research reported a promising efficacy with acceptable toxicity of the addition of pembrolizumab to paclitaxel–carboplatin-based perioperative therapy, showing a high pCR rate of 46.1% and a rate of 82.1% in 1-year OS (74). The trial, Preoperative Anti-PD-1 Antibody Combined With Chemoradiotherapy for Locally Advanced Squamous Cell Carcinoma of Esophageus, was conducted to evaluate the safety and efficacy of a combination of pembrolizumab and the CROSS regimen. Of 65% grade ≥3 AEs, the most common were leukopenia, lymphopenia, anemia, esophagitis, alopecia, and fatigue, which were all acceptable in clinical practices. The addition of pembrolizumab did not lead to the delay of surgery. After the treatment, the pCR reached 55.6%, which was higher than 49% in the CROSS trial and 43.2% in the NEOCRTEC5010 trial (75). The trial, PDL-1 Targeting in Resectable esophageal Cancer (PERFECT), reported the feasibility of atezolizumab combined with the CROSS regimen for locally advanced ESCC. The rate of completion was 83% with no effects on operation interval. 40% of grade ≥3 AEs were observed, of which the most common AEs were anorexia, nausea, and syncope. The rate of immune-related AEs was 16% including 2 for grade 3 rash, 2 for grade 2 colitis/proctitis, and 2 for grade 2 thyroiditis. However, PCR, mOS, and mPFS had no statistical differences compared with a CROSS regimen cohort (114 patients) (76).

As shown above, current studies suggested neoadjuvant immunotherapy combined with chemo(radio)therapy was feasible. Patients who received such modality had a comparable or even higher pCR compared with conventionally neoadjuvant therapy. Notability, most current results are from phase I/II trials. Whether neoadjuvant immunotherapy combined with chemo(radio)therapy can bring a long survival benefit for patients with LAEC requires adequately valid evidence from phase III trials. The synergistic effects between immunotherapy, chemotherapy, and radiation have been reported. Chemotherapy can either lead to immunosuppression or immune activation, which is related to the change of the composition of the tumor microenvironment like priming or inhibiting the expression of immunosuppressor genes (77). Radiation can help expose the tumor antigen to enhance immune response, while immunotherapy can increase the sensitivity of tumors to radiation. These interactive actions should inspire scientists to focus on the sequence between immunotherapy and conventional chemo(radio)therapy. So far, a trial (NCT03985670) is exploring the effect of the sequence of toripalimab and chemotherapy (paclitaxel/cisplatin, sequential or concurrent) on pCR. Initial results showed a fivefold discrepancy in DFS between the two settings (78). Moreover, as the synergistic effects between immunotherapy and radiation, the modality of radiation including dose and fraction may be changed since its role is no longer to lead to cytotoxicity alone but also to assist the immune system. The interactive actions between radiation and tumor microenvironment should be paid attention to. In addition, the KEYNOTE181 trial showed different efficacy in different populations. Also, some research demonstrated that patients with a higher expression of PD-L1 obtain more benefits. All these raise another question about how to screen the benefit population.

## Summary

At present, neoadjuvant therapy is the mainstay for patients with locally esophageal carcinoma. Based on several landmark trials, nCRT has been confirmed to be superior to surgery alone in R0 resection, survival outcomes, and recurrence. So far, the standard regimens for nCRT are the paclitaxel–carboplatin-based regimen for EAC or ESCC from the CROSS trial and vinorelbine–cisplatin-based regimen for ESCC from the NEOCRTEC5010 trial. nCT is also a kind of strategy for LAECs. Especially in Japan, based on its own studies, nCT with the CF-based regimen is the standard modality for locally advanced ESCC. The DCF regimen may replace the CF regimen as a new standard regimen for nCT based on the results from the JCOG1109 NExT trial recently. For locally advanced EAC, nCT based on the MAGIC regimen or perioperative chemotherapy based on the FLOT regimen are the main strategies. As the evidence from randomized clinical trials is limited, it is not yet clear which of these two treatment modalities is better. Histologically, the OE02 trial demonstrated that the efficacy of nCT based on the CF regimen is irrespective of the histological type as no heterogeneity of treatment effect (*P*=0.81). However, the results from meta-analyses demonstrated that nCT did not improve the survival of patients with ESCC(*P*=0.18) but increased the survival of those with EAC (*P*=0.01) (79). Besides, as mentioned above, the recurrence pattern between these two pathologic tumor types is different. Patients with EAC are more likely to have distant metastasis, while those with ESCC are more prone to local recurrence. Furthermore, compared with EAC, ESCC is more sensitive to radiation. Considering these facts, patients with locally advanced EAC might be prone to receive preoperative or perioperative chemotherapy, while those with locally advanced ESCC might be prone to choose nCRT. However, it is more essential to depend on individual characteristics and the building of hospital technology such as physical conditions, individual tumor characteristics, the prediction of pCR or recurrence, and a multidisciplinary cancer treatment team. Although nCRT has been regarded as the standard treatment for LAECs, some unsolved clinical problems exist like the optimal regimen, radiation dose, surgical intervals, and a high risk of recurrences. pCR is an important predictor of a good prognosis of patients. However, currently, accurate methods to evaluate pathological response after nCRT are limited. Future studies should focus on the research regarding multiple parameters to predict pCR. Immunotherapy combined with neoadjuvant therapy has shown promising anti-efficacy. For a better synergistic effect, future research should focus on the sequence of immunotherapy and chemo(radio)therapy and biomarkers to the recognized beneficiary population.

## Author Contributions

Conceptualization: RH, ZQ, and EL. Writing – Original Draft Preparation: RH and ZQ. Writing – Review and Editing: YX and EL. Funding Acquisition: YX and EL. All authors read and approved the final manuscript.

## Funding

This work was supported by funding from the Guangdong College Students’ Scientific and Technological Innovation cultivation special fund subsidy project(pdjhb0195; pdjh2020a0218), Cultivation of Guangdong College Students’ Scientific and Technological Innovation (‘Climbing Program’ Special Funds, pdjh2019a0182), the National Undergraduate Training Program for Innovation and Entrepreneurship (201810560037), ‘Young Physician Scientist Cultivation’ Program of Shantou University Medical College-Li Ka Shing Foundation, 2017-2020 (SMLYPSC-01), and the National Natural Science Foundation of China (21907063).

## Conflict of Interest

The authors declare that the research was conducted in the absence of any commercial or financial relationships that could be construed as a potential conflict of interest.

## Publisher’s Note

All claims expressed in this article are solely those of the authors and do not necessarily represent those of their affiliated organizations, or those of the publisher, the editors and the reviewers. Any product that may be evaluated in this article, or claim that may be made by its manufacturer, is not guaranteed or endorsed by the publisher.

## Glossary

**Table:**

HR	hazard ratio
FLOT-AIO, 5-FU	leucovorin, oxaliplatin, and docetaxel (FLOT) versus epirubicin, cisplatin, and 5-FU (ECF) in patients with locally advanced resectable gastric cancer
FLOT	5-fluorouracil, leucovorin, oxaliplatin, docetaxel
mOS	median overall survival
mDFS	median disease-free survival
ESCC	esophageal squamous carcinoma
OS	overall survival
JCOG9907 trial	the Japan Clinical Oncology Group 9907 trial
CF	cisplatin/5-fluorouracil
pCR	pathological complete response
DCF	docetaxel, cisplatin, 5-FU
PFS	progression-free survival
JCOG1109 NExT trial	the Japan Clinical Oncology Group NexT trial
ASCO-GI	American Society of Clinical Oncology Gastrointestinal Cancers Symposium
CROSS trial	the ChemoRadiotherapy for Oesophageal cancer followed by Surgery Study trial
NEOCRTEC5010	Phase III Study of Neo-adjuvant Chemoradiotherapy Foloowed by Surgery for Squamous Cell Esophageal Cancer
mPFS	median progression-free survival
DFS	disease-free survival
OR	odd ratios
ESC	interdisciplinary supportive care
AEs	adverse events
ORR	objective response rate
DoR	duration of response
POET	PreOperative therapy in Esophagogastric adenocarcinoma
NeoRes	Neoadjuvant Chemotherapy Versus Radiochemotherapy for Cancer of the Esophagus or Cardia
CMISG1701	Comparison Between NCRT and NCT Followed by MIE for Treatment of Locally Advanced Resectable ESCC
MIE	minimally invasive esophagectomy
18F-FDG PET(-CT)	Positron emission tomography with 2-deoxy-2-[fluorine-18]fluoro-D-glucose integrated (with computed tomography)
MRI	magnetic resonance imaging
DW-MRI	Diffusion-weighted magnetic resonance imaging
DCE-MRI	Dynamic contrast-enhanced magnetic resonance imaging
preSANO trial	the Surgery As Needed for Oesophageal Cancer trial
KEYNOTE 181	Study of(MK-3475) Versus Investigator’s Choice Standard Therapy for Participants With Advanced Esophageal/Esophagogastric Junction Carcinoma That Progressed After First-Line Therapy
KEYNOTE 590	First-line Esophageal Carcinoma Study With Chemo cs. Chemo Plus Pembrolizumab
ATTRACTION-3	Study of Nivolumab in Unresectable Advanced or Recurrent Esophageal Cancer
ESCORT	Study of SHR-1210 Versus Investigator’s Choice Standard Therapy for Participants With Advanced Esophageal Cancer
ESCORT-1^st^	Study of SHR-1210 in Combination With Chemotherapy in Advanced Esophagea Cancer
PALACE-1	Preoperative Anti PD-1 Antibody Combined With Chemoradiotherapy for Locally Advanced Squamous Cell Carcinoma of Esophageus
PERFECT	PDL-1 targeting in resectable esophageal cancer
XELOX	capecitabine, oxaliplatin
FOLFOX	leucovorin, fluorouracil, oxaliplatin
