# Supplementary material for: Neoadjuvant Therapy for Locally Advanced Esophageal Cancers
Source: Front Oncol. 2022 Apr 7;12:734581. doi: 10.3389/fonc.2022.734581 (PMC9021527; doi:10.3389/fonc.2022.734581)
Supplement: Supplementary file 1 [file Table_1.docx]

| **Supplementary table 1. Clinical trials regarding immunotherapy combined with chemo(radio)therapy** | | | | | | | | |
| --- | --- | --- | --- | --- | --- | --- | --- | --- |
| **Clinical trials** | **Phase** | **Size** | **Drug** | **Conditions** | **Interventions** | **Primary endpoints** | **Secondary endpoints** | **Time** |
| NCT04767295 | II | 28 | Camrelizumb | ESCC | 2 cycles of camrelizumab, albumin paclitaxel, carboplatin + surgery | ORR,PFS,OS,DCR,pCR | None | 2021-2023 |
| NCT04506138 | I/II | 46 | Camrelizumb | ESCC | Camrelizumab, paclitaxel, carboplatin + surgery | pCR,MPR | OS, EFS | 2020-2025 |
| NCT04225364 | II | 56 | Camrelizumb | ESCC | 2 cycles of camrelizumab, albumin-bound paclitaxel, cisplatin + surgery | pCR | ORR, PFS, OS | 2020-2021 |
| NCT03200691 | II | 21 | Camrelizumb | ESCC | 2 cycles of camrelizumab concurrently with 40Gy+surgery | pCR | DFS, AE | 2018-2021 |
| NCT03917966 | II | 40 | Camrelizumb | ESCC | 2 cycles of camrelizumab, docetaxel, nedaplatin + surgery | ORR, MPR | pCR,OS,DFS,Lymph node derating rate,R0 | 2020-2022 |
| NCT02998268 | II | 46 | Pembrolizumab | EAC; AC of GEJ | Cohort1: Induction chemotherapy (taxol/ carboplatin) + nCRT(taxol/carboplatin/pembrolizumab) + surgery+pembrolizumab Cohort2: Induction chemotherapy (taxol/ carboplatin/ pembrolizumab) + nCRT (taxol/ carboplatin/ pembrolizumab) + surgery + pembrolizumab | DFS | pCR, R0, Median survival rates,1-year OS,Reduction of local immune infiltration | 2017-2021 |
| NCT03792347 | IB | 20 | Pembrolizumab | ESCC | CROSS regimen concurrently with pembrolizumab,41.4Gy+surgery | Safety | Feasibility; pCR; Radiographic response | 2019-2020 |
| NCT04435197 | II | 143 | Pembrolizumab | ESCC | CROSS regimen concurrently with pembrolizumab,41.4Gy+surgery | pCR | 3-year DFS; OS | 2020-2025 |
| NCT04389177 | II | 50 | Pembrolizumab | ESCC | 3 cycles of pembrolizumab, paclitaxel, cisplatin + surgery | MPR | ORR, DFS, OS, Lymph node derating rate, R0 | 2020-2022 |
| NCT04807673 | III | 342 | Pembrolizumab | ESCC) | Arm1: 3 cycles of pembrolizumab, paclitaxel , cisplatin+ surgery + permbrolizumab Arm2: 5 cycles of paclitaxel, cisplatin concurrently with 41.4Gy +surgery | EFS | OS, DFS, MPR, ORR, pCR, AE, QoL | 2021-2028 |
| NCT02844075 | II | 18 | Pembrolizumab | ESCC | nCRT (taxol/ carboplatin, 44.1Gy) concurrently with pembrolizumab + surgery + pembrolizumab | pCR | DFS; OS; Pathologic response rate; AE | 2017-2022 |
| NCT03064490 | II | 38 | Pembrolizumab | EC, AC of stomach, GEJ | 3 cycles of pembrolizumab concurrently with CROSS regimen, 45Gy + surgery + 3 cycles of Pembrolizumab | pCR | Safety | 2017-2023 |
| NCT04437212 | II | 20 | Toripalimab | ESCC | 5 cycles of paclitaxel, cisplatin+ 2 cycles of toripalimab (240mg) + surgery + 4 cycles of toripalimab (240mg) | MPR | 2-year DFS/OS, Treatment-related AE | 2020-2023 |
| NCT04644250 | II | 32 | Toripalimab | ESCC | 5 cycles of paclitaxel liposome, carboplatin (AUC=2) concurrently with 3 cycles of toripalimab, 41.4Gy + surgery | pCR | Treatment-related AE, 2-year OS/DFS, MPR, ORR, R0 | 2022-2024 |
| NCT04280822 | III | 400 | Toripalimab | ESCC | Arm1: 2 cycles of paclitaxel, cisplatin, toripalimab + surgery Arm2: 2 cycles of paclitaxel, cisplatin +surgery | EFS | pCR, DFS, OS, ORR, R0, MPR | 2020-2028 |
| NCT04848753 | III | 500 | Toripalimab | ESCC | Arm1: Toripalimab combined with cisplatin and paclitaxel + surgery Arm2: Placebo combined with cisplatin and paclitaxel + surgery | EFS | pCR, EFS, OS, 1/3/5-year OS | 2021-2026 |
| NCT04177797 | II | 20 | Toripalimab | ESCC | 2 cycles of toripalimab, paclitaxe), carboplatin + surgery | pCR | DCR, ORR, AE | 2020-2021 |
| NCT04888403 | II | 45 | Toripalimab | ESCC | 1 cycle of toripalimab, ablumin palictaxel, nedaplatin concurrently with 41.4Gy+surgery | pCR | MPR, DFS, R0, Postoperative complications rate | 2020-2022 |
| NCT04177875 | II | 44 | Toripalimab | EC | 2 cycles of teripalimab, docetaxel /albumin-bound paclitaxel, cisplatin concurrently with 40Gy+surgery | MPR, ORR | 2-year DFS, mDFS, mOS, AE | 2019-2022 |
| NCT04006041 | II | 44 | Toripalimab | ESCC | 4 cycles of paclitaxel, cisplatin and 2 cycles of toripalimabconcurrently with 44Gy + surgery | pCR | 2-year OS/DFS, AE, R0, Perioperative complication rate,Perioperative mortality | 2019-2020 |
| NCT03985670 | II | 30 | Teripalimab | ESCC | Arm1: Teripalimab concurrently with paclitaxel, cisplatin + surgery Arm2: Paclitaxel, cisplatin sequently with teripalimab + surgery | pCR | AE, DFS | 2019-2023 |
| NCT04804696 | II | 53 | Toripalimab | ESCC | Toripalimab paclitaxel and cisplatin | pCR | ORR, R0, MPR, DFS, OS, AE | 2021-2024 |
| NCT03044613 | I | 32 | Nivolumab | EAC | Arm 1: 2 cycles of nivolumab sequently with CROSS regimen concurrently with 41.4Gy+surgery Arim2: 2 cycles of nivolumab, relatlimab sequently with CROSS regimen concurrently with 41.4Gy + surgery | Treatment-related AE | Feasibility, pCR, Approximate quantitation of infused nivolumab bound to PD-1 in the peripheral blood and within the resected tumor and lymph node specimens,Expression of selected immune markers, OS, RFS | 2017-2024 |
| NCT03914443 | I | 36 | Nivolumab | EC | Cohort1: nivolumab,5-FU, cisplatin + surgery Cohort2: nivolumab),5-FU, cisplatin + surgery Cohort3: nivolumab,5-FU, cisplatin, DTX + surgery Cohort4: nivolumab,5-FU, cisplatin, DTX + surgery | Rate of DLTs | RR, pCR, R0, Completion rate, AE, PFS, OS | 2019-2022 |
| NCT03987815 | II | 20 | Nivolumab | ESCC | Nivolumab for maximum of 3 cycles + surgery | MPR | ORR, PET-CT response, PFS, OS | 2019-2022 |
| NCT03544736 | I/II | 30 | Nivolumab | EC; AC of GEJ | Chort1: Nivolumab concurrently with 20-50Gy+surgery Chort2: Definitive chemoradiotherapy (paclitaxel. Carboplatin) combined with paclitaxel carboplatin, nivolumab (240/480mg during/after RT) Chort3: Paclitaxel, carboplatin, nivoluma concurrently with 41.4Gy + surgery+nivolumab (48mg) | Treatment-related AE | Resposne, OS, PFS, QoL | 2018-2040 |
| NCT03278626 | I/II | 10 | Nivolumab | ESCC | Nivolumab,paclitaxel, carboplatin concurrently with 50.4Gy + surgery | UT | None | 2017-2021 |
| NCT03946969 | I/II | 40 | Sintilimab | ESCC | 2 cycles of sintilimab, liposomal paclitaxel, cisplatin, S-1 + surgery | Treatment-emergent AE | MPR, R0, ORR, RFS, OS | 2019-2022 |
| NCT03940001 | I | 20 | Sintilimab | ESCC | Sintilimab, paclitaxel), carboplatin (AUC=2) concurrently with 41.4Gy+surgery | Toxicity, pCR, MPR | None | 2019-2022 |
| NCT04625543 | II | 100 | Sintilimab | EC | Arm1: 2 cycles of paclitaxel, cisplatin, sintilimab + surgery Arm2: 2 cycles of paclitaxel, cisplatin+ surgery | MPR | ORR,2-year PFS, AE | 2020-2023 |
| NCT04548440 | II | 50 | Sintilimab | ESCC | 2-4 cycles of sintilimab, albumin-bound paclitaxel, cisplatin+ surgery | R0 | pCR, PFS, Tumor regression rate, Relapse rate, OS, AE | 2020-2022 |
| NCT04776590 | II | 30 | Tislelizumab | ESCC | Albumin-bound paclitaxel, carboplatin, tislelizumab concurrently with 41.4Gy+surgery | pCR | DFS, OS | 2021-2024 |
| NCT02962063 | I/II | 78 | Durvalumab | EAC; AC of GEJ | 2 cycles of mFOLFOX6+1 cycle of durvalumab,tremelimumab+50.4Gy cocurrently with [for PET responsders: oxaliplatin,5-FU or capecitabine;for PET non-responders: 5 cycles of carboplatin, paclitaxel+surgery | UT, pCR | OS | 2016-2023 |
| NCT04159974 | II | 56 | Durvalumab | EAC; AC of GEJ | Arm1: neoadjuvant therapy: durvalumab for 1year+surgery Arm2: Adjuvant therapy: durvalumab, tremelimumab | Safety and efficacy | BOR, PFS, DFS, OS, QoL | 2019-2024 |
| NCT04568200 | II | 60 | Durvalumab | ESCC | Arm1: Duralumab and neoadjuvant therapy (paclitaxel; carboplatin; with/without 41.4Gy) + surgery Arm2: Normal saline and neoadjuvant therapy(paclitaxel, carboplatin with/without 41.4Gy)+surgery | Tumor response, Pathological response | Completion rate of treatment, Toxicity, Withdrawal/delay rate from surgery due to durvalumab related complications, Postoperative complications, PFS, OS | 2020-2023 |
| NCT03087864 | II | 40 | Atezolizumab | EAC; AC of GEJ | Atezolizumab carboplatin , paclitaxel concurrently with 41.4Gy + surgery | completion rate of the treatment | Toxicity, Completion rate, Withdrawal/delay rate from surgery due to atezolizumab related complications, Postoperative complications, Pathological response, R0, PFS, OS | 2017-2019 |
| NCT03784326 | I | 30 | Atezolizumab | EAC; AC of GEJ | 3 cycles of oxaliplatin, fluorouracil, atezolizumab+surgery+3 cycles of oxaliplatin, fluorouracil, atezolizumab+4 cycles of atezolizumab | pCR | DFS, OS, Changes in tumor stroma profile, Toxicity, Tumor regression | 2019-2021 |
| NCT03399071 | II | 40 | Atezolizumab | EAC, AC of stomach, GEJ | Avelumab(10mg/kg) followed by FLOT regimen+surgery | pCR | AE; Radiological response rate; mPFS; mOS | 2017-2025 |
| NCT03490292 | I/II | 24 | Atezolizumab | EC | Part1: 3 cycles of paclitaxel, carboplatin , avelumab+ surgery + 6 cycles of Avelumab Part2: the proposed treatment based on Part1 | Part1: Dose limiting Toxicity Part2: pCR | AE, Completion of the treatment, DFS, Surgical complications, R0 | 2018-2024 |
| *ESCC* esophageal squamous carcinoma, *EAC* esophageal adenocarcinoma, *AC* adenocarcinoma, *GEJ* gastroesophageal-junction, *EC* esophageal cancer, *ORR* objective response rate, *PFS* progression free survival, *OS* overall survival, *DCR* disease control rate, *pCR* pathological complete response, *MPR* major pathological response, *EFS* event free survival, *QoL* quality of life, *DCR* disease control rate, *DLT* dose limiting toxicities, *RR* response rate, *UT* unacceptable toxicity, *BOR* best objective response, *CROSS* 5 cycles of paclitaxel(50mg/m2) and carboplatin(2mg/AUC) on days1,8,15,22 and 29, *mFOLFOX6* bolus 5-FU:400mg/m2;leucovorin:400mg/m2;oxaliplatin:70-85 mg/m2 and infusional 5-FU:1200mg/m2/day, q2w, *FLOT* Oxaliplatin:85mg/m2 on day1;Docetaxel:50mg/m2 on day1;Fluorouracil:2600mg/m2 | | | | | | | | |
